# Bis(4-meth­oxy­benzyl­ammonium) dihydrogen diphosphate

**DOI:** 10.1107/S1600536812051616

**Published:** 2013-01-09

**Authors:** Adel Elboulali, Samah Akriche, Salem S. Al-Deyab, Mohamed Rzaigui

**Affiliations:** a Laboratoire de Chimie des Matériaux, Faculté des Sciences de Bizerte, 7021 Zarzouna Bizerte, Tunisia; b Petrochemical Research Chair, College of Science, King Saud, University, Riyadh, Saudi Arabia

## Abstract

In the title compound, 2C_8_H_12_NO^+^·H_2_P_2_O_7_^2−^, the linked PO_4_ groups of the diphosphate anion are almost eclipsed and the P—O—P angle is 134.45 (7)°. In the crystal, infinite ribbons of H_2_P_2_O_7_^2−^ anions propagate in [100], being linked by strong O—H⋯O hydrogen bonds. The 4-meth­oxy­benzyl­ammonium cations bond to the diphosphate chains by N—H⋯O and C—H⋯O links, and are themselves linked by C—H⋯π inter­actions.

## Related literature

For background to diphosphates, see: Ballarini *et al.* (2006 ▶); For inter­molecular inter­actions, see: Brown (1976 ▶); Tiekink & Zukerman-Schpector (2012 ▶). For a related structure, see: Ahmed *et al.* (2006 ▶).
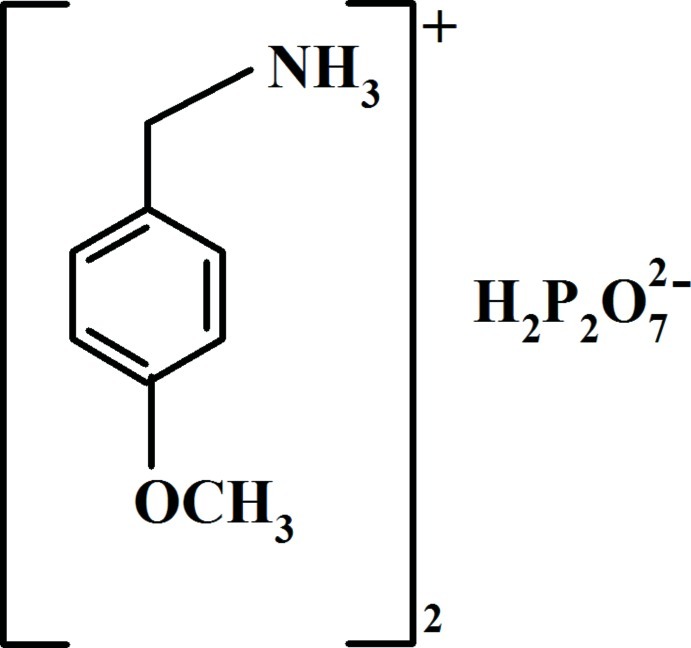


## Experimental

### 

#### Crystal data

2C_8_H_12_NO^+^·H_2_P_2_O_7_^2−^
*M* *_r_* = 452.33
Triclinic, 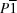

*a* = 9.184 (3) Å
*b* = 6.737 (4) Å
*c* = 17.066 (2) Å
α = 97.61 (2)°
β = 91.39 (4)°
γ = 85.72 (3)°
*V* = 1043.6 (7) Å^3^
*Z* = 2
Ag *K*α radiation
λ = 0.56087 Å
μ = 0.14 mm^−1^
*T* = 296 K
0.30 × 0.25 × 0.17 mm

#### Data collection

Enraf–Nonius CAD-4 diffractometer
12631 measured reflections
10225 independent reflections
5553 reflections with *I* > 2σ(*I*)
*R*_int_ = 0.026
2 standard reflections every 120 min intensity decay: none

#### Refinement

*R*[*F* ^2^ > 2σ(*F* ^2^)] = 0.055
*wR*(*F* ^2^) = 0.143
*S* = 0.98
10225 reflections
268 parameters
H-atom parameters constrained
Δρ_max_ = 0.38 e Å^−3^
Δρ_min_ = −0.51 e Å^−3^

### 

Data collection: *CAD-4 EXPRESS* (Enraf–Nonius, 1994 ▶); cell refinement: *CAD-4 EXPRESS*; data reduction: *XCAD4* (Harms & Wocadlo, 1995 ▶); program(s) used to solve structure: *SHELXS86* (Sheldrick, 2008 ▶); program(s) used to refine structure: *SHELXL97* (Sheldrick, 2008 ▶); molecular graphics: *ORTEP-3* (Farrugia, 2012 ▶) and *DIAMOND* (Brandenburg & Putz, 2005 ▶); software used to prepare material for publication: *WinGX* (Farrugia, 2012 ▶).

## Supplementary Material

Crystal structure: contains datablock(s) I, global. DOI: 10.1107/S1600536812051616/hb7003sup1.cif

Structure factors: contains datablock(s) I. DOI: 10.1107/S1600536812051616/hb7003Isup2.hkl

Supplementary material file. DOI: 10.1107/S1600536812051616/hb7003Isup3.cml

Additional supplementary materials:  crystallographic information; 3D view; checkCIF report

## Figures and Tables

**Table 1:** Hydrogen-bond geometry (Å, °) *Cg*1 and *Cg*2 are the centroids of the C2–C7 and C10–C15 rings, respectively.

*D*—H⋯*A*	*D*—H	H⋯*A*	*D*⋯*A*	*D*—H⋯*A*
O1—H1⋯O6^i^	0.82	1.82	2.6347 (18)	176
O5—H5⋯O2^ii^	0.82	1.75	2.5535 (18)	164
N1—H1*A*⋯O3^iii^	0.89	2.09	2.941 (2)	160
N1—H1*B*⋯O3^ii^	0.89	1.97	2.857 (2)	172
N1—H1*C*⋯O2	0.89	2.03	2.915 (2)	173
N2—H2*B*⋯O6	0.89	2.35	3.156 (2)	151
N2—H2*A*⋯O6^iv^	0.89	1.89	2.734 (2)	157
N2—H2*B*⋯O4	0.89	2.38	3.150 (2)	145
N2—H2*C*⋯O7^i^	0.89	1.85	2.724 (2)	168
C1—H1*D*⋯O7^ii^	0.97	2.49	3.242 (3)	134
C7—H7⋯O2	0.93	2.54	3.195 (2)	127
C16—H16*C*⋯*Cg*1^v^	0.96	2.93	3.73 (7)	142
C8—H8*A*⋯*Cg*2	0.96	2.97	3.72 (7)	137
C1—H1*D*⋯*Cg*2^vi^	0.97	2.90	3.54 (7)	124

Symmetry codes: (i) 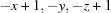; (ii) 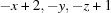; (iii) 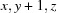; (iv) 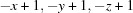; (v) 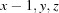; (vi) 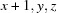.

**Fig. 1.**
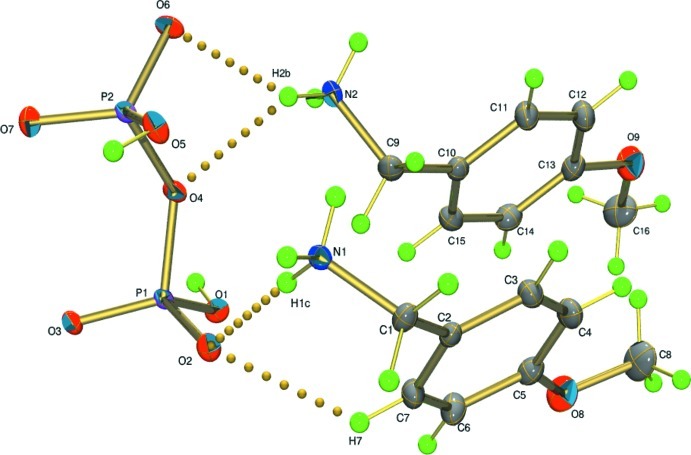
The molecular structure of (I) with displacement ellipsoids drawn at the 30% probability level. Hydrogen bonds are represented as dashed lines.

**Fig. 2.**
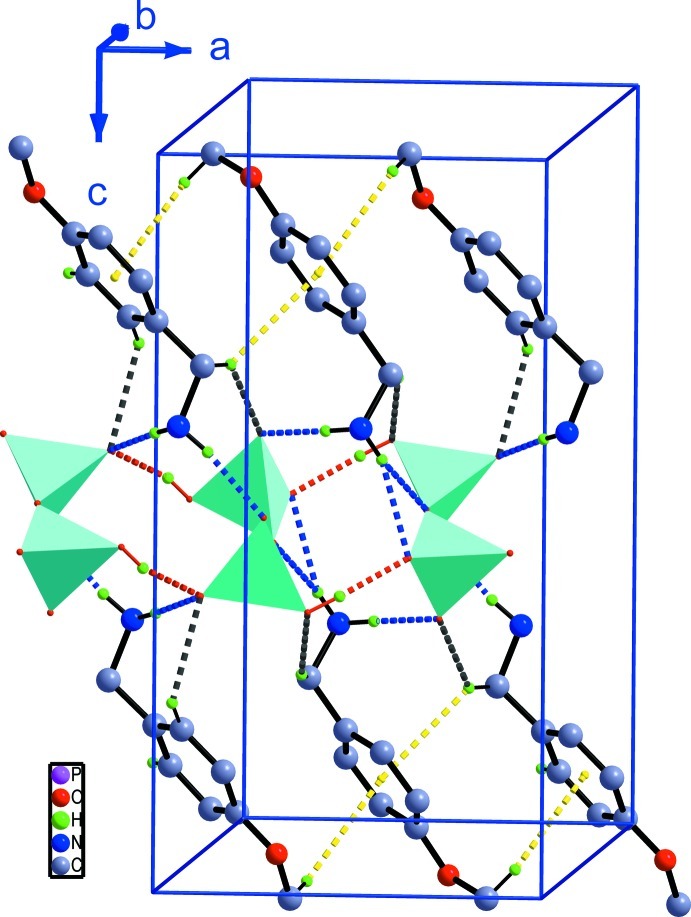
Perspective view of the packing of (I). The H-atoms not involved in H-bonding are omitted.

## supplementary crystallographic information

### Comment

Diphosphates are known to play an important role as catalysts (Ballarini *et al.*, 2006). As part of our studies in this area, we report the synthesis and the crystal structure of the title compound, (I) (Fig. 1).

In this structure, [H_2_P_2_O_7_]^2-^ species are connected by means of strong hydrogen bonds of type O—H···O with O···O distances less than 2.7 Å, limit as recommended by Brown (1976). This infinite sequence forms ribbons extending along *a* axis.

Except the H atoms, the P_2_O_7_ group, has an eclipsed conformation evidenced by the torsion angle O3—P1—P2—O7 = -1.5°. As usually observed for diphosphate groups (Ahmed *et al.*, 2006), there are three different types of P—O distances, the longest one corresponds to the bridging oxygen atom with average value d(P—O4) = 1,608 (1) Å, the intermediate ones are the P—OH bonding [*d*(P1—O1) = 1.566 (1) Å, d(P2—O5) = 1.552 (1) Å], whereas the shortest ones, spreading between 1.474 (1) Å and 1.503 (1) Å are related to the external oxygen atoms. The average values of the P—O distances and O—P—O angles are 1,536 (1) Å and 109,24 (7)° respectively.

The organic cations linked by C–H···π interaction (Tiekink and Zukerman-Schpector, 2012) into chains along *a* axis, are anchored onto successive inorganic ribbons [H_2_P_2_O_7_]_n_^2n-^ through hydrogen bonds of type N—H···O and C—H···O with donor-acceptor distances varying between 2.724 (2) Å and 3.156 (2) Å.

It should be noticed that another diphosphate with the same organic molecule, [4-(OCH_3_)C_6_H_4_CH_2_NH_3_]_4_P_2_O_7_.6H_2_O, has been reported by Ahmed *et al.* (2006). Structure of this hydrated diphosphate is different from that of the non-hydrated one described here. This difference may be explained by the role of water of crystallization as directing structure agent.

### Experimental

An aqueous solution of diphosphoric acid H_4_P_2_O_7_ was first obtained by passing a solution of Na_4_P_2_O_7_ (3 g, 11.2 mmol), through an ion exchange resin (Amberlite IR 120) in its H-state. To 20 ml of this acidic solution (1.5 mmol) cooled to 5°C, a solution of 4-methoxybenzylamine (3 mmol) in ethanol (3 mL),was added drop by drop with slow stirring. The obtained solution was slowly evapored at room temperature until crystallization of colourless prisms.

### Refinement

All H atoms attached to C and N atoms were fixed geometrically and treated as riding, with C—H = 0.97 Å and N—H = 0.89 Å and with *U*_iso_(H) = 1.2Ueq(C or N).The water H atoms were refined using restraints [O— H = 0.85 (1) A °, H···H = 1.44 (2) A ° and *U*_iso_(H) = 1.5Ueq(O)].

### Crystal data

**Table:**

2C_8_H_12_NO^+^·H_2_O_7_P_2_^2^^−^	*Z* = 2
*M**_r_* = 452.33	*F*(000) = 476
Triclinic, *P*1	*D*_x_ = 1.439 Mg m^−^^3^
*a* = 9.184 (3) Å	Ag *K*α radiation, λ = 0.56087 Å
*b* = 6.737 (4) Å	Cell parameters from 25 reflections
*c* = 17.066 (2) Å	θ = 9–11°
α = 97.61 (2)°	µ = 0.14 mm^−^^1^
β = 91.39 (4)°	*T* = 296 K
γ = 85.72 (3)°	Prism, colorless
*V* = 1043.6 (7) Å^3^	0.30 × 0.25 × 0.17 mm

### Data collection

**Table:**

Enraf–Nonius CAD-4 diffractometer	*R*_int_ = 0.026
Radiation source: fine-focus sealed tube	θ_max_ = 28.0°, θ_min_ = 2.0°
Graphite monochromator	*h* = −15→15
non–profiled ω scans	*k* = −11→11
12631 measured reflections	*l* = −4→28
10225 independent reflections	2 standard reflections every 120 min
5553 reflections with *I* > 2σ(*I*)	intensity decay: none

### Refinement

**Table:**

Refinement on *F*^2^	Primary atom site location: structure-invariant direct methods
Least-squares matrix: full	Secondary atom site location: difference Fourier map
*R*[*F*^2^ > 2σ(*F*^2^)] = 0.055	Hydrogen site location: inferred from neighbouring sites
*wR*(*F*^2^) = 0.143	H-atom parameters constrained
*S* = 0.98	*w* = 1/[σ^2^(*F*_o_^2^) + (0.0674*P*)^2^] where *P* = (*F*_o_^2^ + 2*F*_c_^2^)/3
10225 reflections	(Δ/σ)_max_ = 0.001
268 parameters	Δρ_max_ = 0.38 e Å^−^^3^
0 restraints	Δρ_min_ = −0.51 e Å^−^^3^

### Special details

**Table:**

Geometry. All e.s.d.'s (except the e.s.d. in the dihedral angle between two l.s. planes) are estimated using the full covariance matrix. The cell e.s.d.'s are taken into account individually in the estimation of e.s.d.'s in distances, angles and torsion angles; correlations between e.s.d.'s in cell parameters are only used when they are defined by crystal symmetry. An approximate (isotropic) treatment of cell e.s.d.'s is used for estimating e.s.d.'s involving l.s. planes.
Refinement. Refinement of *F*^2^ against ALL reflections. The weighted *R*-factor *wR* and goodness of fit *S* are based on *F*^2^, conventional *R*-factors *R* are based on *F*, with *F* set to zero for negative *F*^2^. The threshold expression of *F*^2^ > σ(*F*^2^) is used only for calculating *R*-factors(gt) *etc*. and is not relevant to the choice of reflections for refinement. *R*-factors based on *F*^2^ are statistically about twice as large as those based on *F*, and *R*- factors based on ALL data will be even larger.

### Fractional atomic coordinates and isotropic or equivalent isotropic displacement parameters (Å^2^)

**Table:**

	*x*	*y*	*z*	*U*_iso_*/*U*_eq_	
P1	0.77375 (4)	−0.16843 (5)	0.42717 (2)	0.02283 (9)	
P2	0.71445 (4)	0.14070 (5)	0.56549 (2)	0.02389 (9)	
O1	0.65658 (11)	−0.21336 (18)	0.36026 (7)	0.0321 (2)	
H1	0.5815	−0.2428	0.3796	0.048*	
O2	0.90518 (11)	−0.09884 (18)	0.39193 (7)	0.0347 (3)	
O3	0.80044 (12)	−0.33687 (15)	0.47500 (7)	0.0309 (2)	
O4	0.69102 (12)	0.02214 (16)	0.47816 (7)	0.0327 (3)	
O5	0.85320 (11)	0.25556 (17)	0.55945 (8)	0.0361 (3)	
H5	0.9218	0.2006	0.5819	0.054*	
O6	0.58394 (11)	0.29007 (16)	0.57185 (7)	0.0341 (3)	
O7	0.73241 (13)	−0.00463 (17)	0.62306 (7)	0.0359 (3)	
O8	0.65303 (18)	0.1234 (3)	0.06727 (9)	0.0624 (4)	
O9	0.14301 (19)	0.4144 (3)	0.07212 (10)	0.0663 (5)	
N1	0.98380 (14)	0.31496 (19)	0.40133 (8)	0.0301 (3)	
H1A	0.9110	0.4056	0.4165	0.045*	
H1B	1.0568	0.3266	0.4365	0.045*	
H1C	0.9523	0.1923	0.3980	0.045*	
N2	0.45655 (14)	0.30089 (19)	0.39890 (8)	0.0323 (3)	
H2A	0.4183	0.4264	0.4106	0.048*	
H2B	0.5181	0.2707	0.4374	0.048*	
H2C	0.3854	0.2171	0.3941	0.048*	
C1	1.03636 (18)	0.3492 (3)	0.32239 (10)	0.0365 (4)	
H1D	1.1277	0.2697	0.3113	0.044*	
H1E	1.0554	0.4895	0.3242	0.044*	
C2	0.92910 (18)	0.2956 (3)	0.25659 (10)	0.0327 (3)	
C3	0.8548 (2)	0.4405 (3)	0.21786 (12)	0.0419 (4)	
H3	0.8676	0.5751	0.2348	0.050*	
C4	0.7619 (2)	0.3905 (3)	0.15447 (12)	0.0473 (5)	
H4	0.7144	0.4906	0.1286	0.057*	
C5	0.7401 (2)	0.1919 (3)	0.12986 (11)	0.0443 (4)	
C6	0.8102 (3)	0.0456 (3)	0.16945 (12)	0.0507 (5)	
H6	0.7930	−0.0885	0.1543	0.061*	
C7	0.9056 (2)	0.0964 (3)	0.23126 (11)	0.0447 (4)	
H7	0.9548	−0.0041	0.2562	0.054*	
C8	0.5843 (3)	0.2674 (5)	0.02229 (15)	0.0744 (8)	
H8A	0.5198	0.3591	0.0555	0.112*	
H8B	0.5293	0.2004	−0.0205	0.112*	
H8C	0.6572	0.3402	0.0015	0.112*	
C9	0.53647 (18)	0.2817 (3)	0.32319 (11)	0.0395 (4)	
H9A	0.5868	0.1492	0.3133	0.047*	
H9B	0.6091	0.3801	0.3271	0.047*	
C10	0.43368 (18)	0.3132 (3)	0.25591 (11)	0.0360 (4)	
C11	0.3878 (2)	0.5050 (3)	0.23911 (12)	0.0465 (5)	
H11	0.4236	0.6163	0.2695	0.056*	
C12	0.2903 (3)	0.5326 (3)	0.17823 (13)	0.0526 (5)	
H12	0.2606	0.6620	0.1682	0.063*	
C13	0.2364 (2)	0.3700 (3)	0.13206 (12)	0.0455 (4)	
C14	0.2793 (2)	0.1798 (3)	0.14782 (12)	0.0491 (5)	
H14	0.2433	0.0692	0.1171	0.059*	
C15	0.3766 (2)	0.1526 (3)	0.20981 (12)	0.0445 (4)	
H15	0.4038	0.0231	0.2205	0.053*	
C16	0.0827 (3)	0.2508 (4)	0.02453 (16)	0.0760 (8)	
H16A	0.1601	0.1615	0.0004	0.114*	
H16B	0.0210	0.3002	−0.0159	0.114*	
H16C	0.0263	0.1799	0.0569	0.114*	

### Atomic displacement parameters (Å^2^)

**Table:**

	*U*^11^	*U*^22^	*U*^33^	*U*^12^	*U*^13^	*U*^23^
P1	0.01629 (14)	0.02364 (17)	0.0285 (2)	−0.00016 (12)	−0.00173 (13)	0.00439 (14)
P2	0.01804 (15)	0.02244 (16)	0.0311 (2)	−0.00183 (12)	−0.00096 (14)	0.00320 (14)
O1	0.0211 (5)	0.0425 (6)	0.0319 (6)	−0.0046 (4)	−0.0042 (4)	0.0013 (5)
O2	0.0191 (4)	0.0422 (6)	0.0458 (7)	−0.0045 (4)	0.0003 (5)	0.0157 (5)
O3	0.0299 (5)	0.0262 (5)	0.0373 (7)	0.0021 (4)	−0.0002 (5)	0.0096 (5)
O4	0.0319 (5)	0.0297 (5)	0.0337 (6)	0.0072 (4)	−0.0070 (5)	−0.0003 (5)
O5	0.0195 (5)	0.0337 (6)	0.0582 (8)	−0.0066 (4)	−0.0068 (5)	0.0160 (5)
O6	0.0200 (4)	0.0297 (5)	0.0504 (8)	0.0015 (4)	0.0020 (5)	−0.0009 (5)
O7	0.0371 (6)	0.0362 (6)	0.0371 (7)	−0.0085 (5)	−0.0051 (5)	0.0124 (5)
O8	0.0640 (10)	0.0773 (11)	0.0443 (9)	−0.0109 (8)	−0.0196 (8)	0.0029 (8)
O9	0.0745 (11)	0.0750 (11)	0.0490 (10)	−0.0068 (9)	−0.0228 (8)	0.0100 (8)
N1	0.0280 (6)	0.0303 (6)	0.0313 (7)	0.0010 (5)	−0.0034 (5)	0.0038 (5)
N2	0.0325 (6)	0.0291 (6)	0.0364 (8)	−0.0037 (5)	−0.0072 (6)	0.0090 (6)
C1	0.0319 (8)	0.0474 (9)	0.0313 (9)	−0.0057 (7)	0.0016 (7)	0.0071 (7)
C2	0.0343 (8)	0.0377 (8)	0.0265 (8)	−0.0014 (6)	0.0024 (6)	0.0059 (7)
C3	0.0450 (10)	0.0372 (9)	0.0432 (11)	0.0004 (7)	−0.0025 (8)	0.0070 (8)
C4	0.0459 (10)	0.0512 (11)	0.0445 (12)	0.0066 (8)	−0.0079 (9)	0.0121 (9)
C5	0.0417 (9)	0.0587 (12)	0.0319 (10)	−0.0050 (8)	−0.0020 (8)	0.0037 (9)
C6	0.0744 (14)	0.0412 (10)	0.0369 (11)	−0.0123 (9)	−0.0061 (10)	0.0036 (8)
C7	0.0614 (12)	0.0396 (9)	0.0331 (10)	0.0004 (8)	−0.0048 (9)	0.0085 (8)
C8	0.0625 (15)	0.109 (2)	0.0515 (15)	−0.0036 (14)	−0.0244 (12)	0.0165 (15)
C9	0.0296 (7)	0.0436 (9)	0.0453 (11)	0.0017 (7)	0.0023 (7)	0.0077 (8)
C10	0.0348 (8)	0.0395 (8)	0.0341 (9)	−0.0020 (7)	0.0033 (7)	0.0067 (7)
C11	0.0615 (12)	0.0386 (9)	0.0396 (11)	−0.0078 (8)	−0.0079 (9)	0.0058 (8)
C12	0.0701 (14)	0.0441 (10)	0.0449 (12)	−0.0030 (10)	−0.0107 (10)	0.0126 (9)
C13	0.0470 (10)	0.0570 (11)	0.0328 (10)	−0.0039 (9)	−0.0013 (8)	0.0074 (9)
C14	0.0541 (11)	0.0489 (11)	0.0431 (12)	−0.0122 (9)	−0.0006 (9)	−0.0028 (9)
C15	0.0494 (10)	0.0379 (9)	0.0459 (12)	−0.0026 (8)	0.0020 (9)	0.0043 (8)
C16	0.0716 (17)	0.095 (2)	0.0559 (16)	−0.0066 (15)	−0.0233 (13)	−0.0054 (14)

### Geometric parameters (Å, º)

**Table:**

P1—O3	1.4860 (13)	C3—C4	1.383 (3)
P1—O2	1.4942 (12)	C3—H3	0.9300
P1—O1	1.5656 (13)	C4—C5	1.376 (3)
P1—O4	1.6042 (13)	C4—H4	0.9300
P2—O7	1.4744 (13)	C5—C6	1.380 (3)
P2—O6	1.5028 (12)	C6—C7	1.379 (3)
P2—O5	1.5517 (12)	C6—H6	0.9300
P2—O4	1.6126 (12)	C7—H7	0.9300
O1—H1	0.8200	C8—H8A	0.9600
O5—H5	0.8200	C8—H8B	0.9600
O8—C5	1.370 (2)	C8—H8C	0.9600
O8—C8	1.418 (3)	C9—C10	1.495 (3)
O9—C13	1.368 (2)	C9—H9A	0.9700
O9—C16	1.419 (3)	C9—H9B	0.9700
N1—C1	1.494 (2)	C10—C15	1.380 (3)
N1—H1A	0.8900	C10—C11	1.393 (3)
N1—H1B	0.8900	C11—C12	1.377 (3)
N1—H1C	0.8900	C11—H11	0.9300
N2—C9	1.487 (2)	C12—C13	1.378 (3)
N2—H2A	0.8900	C12—H12	0.9300
N2—H2B	0.8900	C13—C14	1.371 (3)
N2—H2C	0.8900	C14—C15	1.390 (3)
C1—C2	1.501 (2)	C14—H14	0.9300
C1—H1D	0.9700	C15—H15	0.9300
C1—H1E	0.9700	C16—H16A	0.9600
C2—C3	1.380 (2)	C16—H16B	0.9600
C2—C7	1.386 (3)	C16—H16C	0.9600
			
O3—P1—O2	116.25 (7)	O8—C5—C6	115.48 (19)
O3—P1—O1	112.16 (7)	C4—C5—C6	119.39 (18)
O2—P1—O1	108.81 (7)	C7—C6—C5	120.56 (19)
O3—P1—O4	110.72 (7)	C7—C6—H6	119.7
O2—P1—O4	107.92 (8)	C5—C6—H6	119.7
O1—P1—O4	99.63 (6)	C6—C7—C2	120.72 (18)
O7—P2—O6	118.73 (8)	C6—C7—H7	119.6
O7—P2—O5	112.51 (7)	C2—C7—H7	119.6
O6—P2—O5	108.43 (7)	O8—C8—H8A	109.5
O7—P2—O4	109.41 (7)	O8—C8—H8B	109.5
O6—P2—O4	101.55 (7)	H8A—C8—H8B	109.5
O5—P2—O4	104.81 (8)	O8—C8—H8C	109.5
P1—O1—H1	109.5	H8A—C8—H8C	109.5
P1—O4—P2	134.45 (7)	H8B—C8—H8C	109.5
P2—O5—H5	109.5	N2—C9—C10	110.84 (14)
C5—O8—C8	117.59 (19)	N2—C9—H9A	109.5
C13—O9—C16	117.15 (19)	C10—C9—H9A	109.5
C1—N1—H1A	109.5	N2—C9—H9B	109.5
C1—N1—H1B	109.5	C10—C9—H9B	109.5
H1A—N1—H1B	109.5	H9A—C9—H9B	108.1
C1—N1—H1C	109.5	C15—C10—C11	117.43 (18)
H1A—N1—H1C	109.5	C15—C10—C9	120.98 (17)
H1B—N1—H1C	109.5	C11—C10—C9	121.55 (17)
C9—N2—H2A	109.5	C12—C11—C10	121.12 (18)
C9—N2—H2B	109.5	C12—C11—H11	119.4
H2A—N2—H2B	109.5	C10—C11—H11	119.4
C9—N2—H2C	109.5	C11—C12—C13	120.5 (2)
H2A—N2—H2C	109.5	C11—C12—H12	119.8
H2B—N2—H2C	109.5	C13—C12—H12	119.8
N1—C1—C2	112.96 (14)	O9—C13—C14	124.96 (19)
N1—C1—H1D	109.0	O9—C13—C12	115.6 (2)
C2—C1—H1D	109.0	C14—C13—C12	119.42 (19)
N1—C1—H1E	109.0	C13—C14—C15	119.92 (18)
C2—C1—H1E	109.0	C13—C14—H14	120.0
H1D—C1—H1E	107.8	C15—C14—H14	120.0
C3—C2—C7	117.96 (17)	C10—C15—C14	121.60 (19)
C3—C2—C1	121.67 (16)	C10—C15—H15	119.2
C7—C2—C1	120.33 (16)	C14—C15—H15	119.2
C2—C3—C4	121.69 (18)	O9—C16—H16A	109.5
C2—C3—H3	119.2	O9—C16—H16B	109.5
C4—C3—H3	119.2	H16A—C16—H16B	109.5
C5—C4—C3	119.64 (18)	O9—C16—H16C	109.5
C5—C4—H4	120.2	H16A—C16—H16C	109.5
C3—C4—H4	120.2	H16B—C16—H16C	109.5
O8—C5—C4	125.13 (19)		
			
O3—P1—O4—P2	47.66 (13)	C5—C6—C7—C2	2.2 (3)
O2—P1—O4—P2	−80.60 (12)	C3—C2—C7—C6	−0.3 (3)
O1—P1—O4—P2	165.91 (11)	C1—C2—C7—C6	−178.26 (18)
O7—P2—O4—P1	−48.92 (13)	N2—C9—C10—C15	−94.9 (2)
O6—P2—O4—P1	−175.23 (10)	N2—C9—C10—C11	82.7 (2)
O5—P2—O4—P1	71.95 (12)	C15—C10—C11—C12	−0.7 (3)
N1—C1—C2—C3	110.56 (19)	C9—C10—C11—C12	−178.38 (19)
N1—C1—C2—C7	−71.6 (2)	C10—C11—C12—C13	−0.4 (4)
C7—C2—C3—C4	−1.4 (3)	C16—O9—C13—C14	2.2 (3)
C1—C2—C3—C4	176.49 (18)	C16—O9—C13—C12	−178.6 (2)
C2—C3—C4—C5	1.2 (3)	C11—C12—C13—O9	−178.5 (2)
C8—O8—C5—C4	2.6 (3)	C11—C12—C13—C14	0.8 (3)
C8—O8—C5—C6	−177.0 (2)	O9—C13—C14—C15	179.08 (19)
C3—C4—C5—O8	−178.81 (19)	C12—C13—C14—C15	−0.1 (3)
C3—C4—C5—C6	0.7 (3)	C11—C10—C15—C14	1.3 (3)
O8—C5—C6—C7	177.14 (19)	C9—C10—C15—C14	179.06 (18)
C4—C5—C6—C7	−2.4 (3)	C13—C14—C15—C10	−0.9 (3)

### Hydrogen-bond geometry (Å, º)

Cg1 and Cg2 are the centroids of the C2–C7 and C10–C15 rings, respectively.

**Table:**

*D*—H···*A*	*D*—H	H···*A*	*D*···*A*	*D*—H···*A*
O1—H1···O6^i^	0.82	1.82	2.6347 (18)	176
O5—H5···O2^ii^	0.82	1.75	2.5535 (18)	164
N1—H1*A*···O3^iii^	0.89	2.09	2.941 (2)	160
N1—H1*B*···O3^ii^	0.89	1.97	2.857 (2)	172
N1—H1*C*···O2	0.89	2.03	2.915 (2)	173
N2—H2*B*···O6	0.89	2.35	3.156 (2)	151
N2—H2*A*···O6^iv^	0.89	1.89	2.734 (2)	157
N2—H2*B*···O4	0.89	2.38	3.150 (2)	145
N2—H2*C*···O7^i^	0.89	1.85	2.724 (2)	168
C1—H1*D*···O7^ii^	0.97	2.49	3.242 (3)	134
C7—H7···O2	0.93	2.54	3.195 (2)	127
C16—H16*C*···*Cg*1^v^	0.96	2.93	3.73 (7)	142
C8—H8*A*···*Cg*2	0.96	2.97	3.72 (7)	137
C1—H1*D*···*Cg*2^vi^	0.97	2.90	3.54 (7)	124

Symmetry codes: (i) −*x*+1, −*y*, −*z*+1; (ii) −*x*+2, −*y*, −*z*+1; (iii) *x*, *y*+1, *z*; (iv) −*x*+1, −*y*+1, −*z*+1; (v) *x*−1, *y*, *z*; (vi) *x*+1, *y*, *z*.

## References

[bb7] Ahmed, S., Samah, A. & Mohamed, R. (2006). *Acta Cryst.* E**62**, m1796–m1798.

[bb1] Ballarini, N., Cavani, F., Cortelli, C., Ligi, S., Pierelli, F., Trifiro, F., Fumagalli, C., Mazzoni, G. & Monti, T. (2006). *Top. Catal.* **38**, 147–156.

[bb2] Brandenburg, K. & Putz, H. (2005). *DIAMOND* Crystal Impact GbR, Bonn, Germany.

[bb3] Brown, I. D. (1976). *Acta Cryst.* A**32**, 24–31.

[bb4] Enraf–Nonius (1994). *CAD-4 EXPRESS* Enraf–Nonius, Delft, The Netherlands.

[bb5] Farrugia, L. J. (2012). *J. Appl. Cryst.* **45**, 849–854.

[bb6] Harms, K. & Wocadlo, S. (1995). *XCAD4* University of Marburg, Germany.

[bb8] Sheldrick, G. M. (2008). *Acta Cryst.* A**64**, 112–122. 10.1107/S0108767307043930 18156677

[bb9] Tiekink, E. R. T. & Zukerman-Schpector, J. (2012). In *Importance of π-Interactions in Crystal Engineering*, 1st ed. London: Wiley.

